# Fluctuation Analysis: Can Estimates Be Trusted?

**DOI:** 10.1371/journal.pone.0080958

**Published:** 2013-12-09

**Authors:** Bernard Ycart

**Affiliations:** Bernard Ycart Laboratoire Jean Kuntzmann, Univ. Grenoble-Alpes and CNRS UMR 5224, Grenoble, France; Centrum Wiskunde & Informatica (CWI) & The Netherlands Institute for Systems Biology, The Netherlands

## Abstract

The estimation of mutation rates and relative fitnesses in fluctuation analysis is based on the unrealistic hypothesis that the single-cell times to division are exponentially distributed. Using the classical Luria-Delbrück distribution outside its modelling hypotheses induces an important bias on the estimation of the relative fitness. The model is extended here to any division time distribution. Mutant counts follow a generalization of the Luria-Delbrück distribution, which depends on the mean number of mutations, the relative fitness of normal cells compared to mutants, and the division time distribution of mutant cells. Empirical probability generating function techniques yield precise estimates both of the mean number of mutations and the relative fitness of normal cells compared to mutants. In the case where no information is available on the division time distribution, it is shown that the estimation procedure using constant division times yields more reliable results. Numerical results both on observed and simulated data are reported.

## Introduction

The estimation of mutation parameters in cell growth experiments, or *fluctuation analysis*, has been the object of many studies since its introduction by Luria and Delbrück in 1943 [1]: see reviews by Stewart et al. [2], Angerer [3], and Foster [4]. Fluctuation analysis is based on the *Luria-Delbrück* distribution, derived under different assumptions by Lea and Coulson [5] and Bartlett (in the discussion following Armitage [6]). Mandelbrot [7], then Bartlett [8] later generalized the Luria-Delbrück distribution to the differential growth case. Since then, fluctuation analysis with differential growth rates has been advocated by several authors [9]–[13]. As shown in [14], Luria-Delbrück distributions are made of three ingredients:

1. *The mean number of mutations α*, which is the parameter of main interest. It is the product of the individual probability of mutation (also called mutation rate) by the final number of cells. As already remarked by Luria and Delbrück [1], the law of small numbers implies that the random number of mutations that occur during the experiment follows a Poisson distribution with expectation *α*.
2. *The relative fitness ρ* of normal cells to mutants, i.e. the ratio of the exponential growth rate of normal cells to that of mutants. (Growth rate refers here to the constant speed at which the logarithm of a population of cells grows, not to the size increments of individual cells). The time scale does not influence final counts of mutant cells: it may be chosen so that the growth rate of mutants is 1, in which case *ρ* is the exponential growth rate of normal cells. Exponential growth implies that most random mutations occur rather close to the end of the experiment, and more precisely that the time during which a new mutant clone develops has negative exponential distribution with parameter *ρ*.
3. *The random number of cells M(t) in a mutant clone* that develops for a finite time *t*: it depends crucially on the division times of mutants. In the classical Luria-Delbrück model, mutants are supposed to have exponentially distributed division times, which implies that *M*(*t*) follows the geometric distribution with parameter 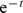 (choosing the time scale so that mutants have unit growth rate).

The first two points can be considered as established facts: they are in accordance with experimental data, and grounded on well known probabilistic results. On the opposite, the hypothesis of exponentially distributed division times is a purely mathematical convenience and does not match experimental observations: as remarked as early as 1932 by Kelly and Rahn [15], [16], actual division times data are unimodal and right-skewed rather than exponential: see [17]. The question investigated here is: which bias on the estimation of the parameters does the exponential distribution hypothesis induce, and how can it be reduced?

The “mathematical convenience” can be challenged. Admittedly, the exponential distribution of division times is the first one under which a closed mathematical expression for the distribution of mutants was obtained. Notwithstanding, it will be shown that a joint estimation procedure for *α* and *ρ* can be implemented whatever the distribution of division times. Moreover if the division times of mutants are supposed to be constant, estimation procedures are exactly as computationally effective as under the exponential hypothesis. Since the pioneering observations of Kelly and Rahn [15] progress in experimental settings, from microscopic observation of single-cell behavior to flow chambers and automated growth analyzers, has fueled many studies on division times and their distributions. Division time data have been fitted by several types of distributions: from Gamma and Log-beta [18], to Log-normal and reciprocal normal [19]: see John [20] and references therein. More recent references include [21]–[24]. There is no such object as “the” distribution of division times; firstly because it would depend not only on the species, strain, experimental conditions, etc., secondly because many different families of distributions can usually fit any given set of observed data. I have chosen three families (Gamma, Log-normal, Inverse Gaussian) and one data set: the historical observations of Kelly and Rahn on Bacterium aerogenes [15]. A maximum likelihood estimation of the parameters on the data led to one particular distribution in each family, that was rescaled to unit growth rate. The three distributions so obtained were considered as realistic and used as benchmarks for extensive Monte-Carlo studies. Samples of size 100 of generalized Luria-Delbrück distributions were repeatedly simulated for different values of *α* and *ρ*, and for each of the three realistic distributions. The main conclusion was that using the classical Luria-Delbrück distribution estimation procedure yields satisfactory results for the estimation of the mean number of mutations *α* but introduces a sizeable bias on the estimation of the relative fitness *ρ*. The estimation procedure that uses constant division times has a negligible bias and a much better precision on *ρ*.

I have developed in R [25] a set of functions that output samples of generalized Luria-Delbrück distributions, compute estimates, confidence regions and p-values for hypothesis testing. These functions have been made available on line: http://www.ljk.imag.fr/membres/Bernard.Ycart/LD/.

## Results

### Simulation experiments

I denote hereafter by 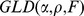 the generalized Luria-Delbrück distribution with parameters *α*, *ρ*, and *F*: it is the distribution of the final number of mutants in a fluctuation analysis experiment, when the mean number of mutations is *α*, the relative fitness of normal cells compared to mutants is *ρ*, and the distribution of mutant division times is *F*. The particular case where division times are exponentially distributed is the classical Luria-Delbrück distribution 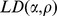. Detailed definitions will be given in the ‘models and methods’ section. In real fluctuation analysis experiments, the actual distribution *F* of division times is unknown. Therefore the question to be answered was the following: if a sample of the generalized Luria-Delbrück distribution 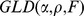 has been produced, and estimates 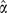 and 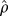 are computed from another division time model than *F*, by how much are these estimates biased, how reliable confidence intervals on *α* and *ρ* can be?

**Figure 1:**
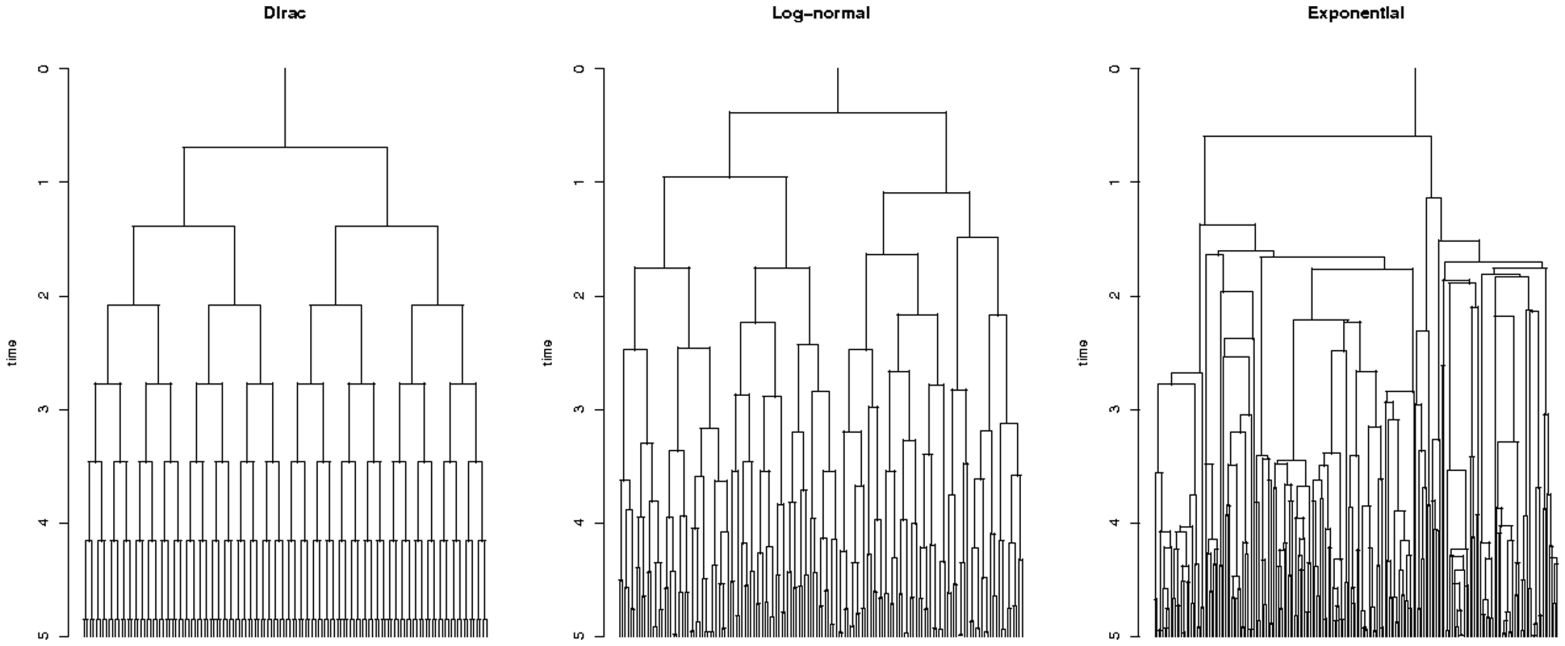
Clones under of Dirac, Log-normal, and Exponential models. The Log-normal distribution has been adjusted on Kelly and Rahn's data. All three distribution have been scaled to have unit growth rates. Clones were simulated up to time 5.

Three distributions were used in simulation procedures: Gamma, Log-normal, and Inverse Gaussian; they were adjusted on Kelly and Rahn's Bacterium aerogenes data. The exact definition of the three distributions is detailed in the ‘models and methods’ section. Two models were considered for estimation: the exponential model (division times follow the negative exponential distribution, i.e. the classical model), and the Dirac model (all division times are equal to the same value). The corresponding distribution functions are denoted by 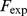 and 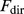. The estimation procedure is explained in the ‘models and methods’ section. Figure 1 represents the evolution of three typical clones, simulated with the Dirac model, the Log-normal model, and the exponential model: the exponential model is much more irregular than observed in practice: see e.g. Figure 5 in [26].

The simulation study consisted in simulating samples of the 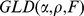, *F* being a Gamma, Log-normal, or Inverse Gaussian distribution, then estimating *α* and *ρ* as if *F* had been 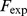 or 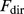. A simulation function for the 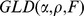 has been included in the R script made available on line. It was used to output 10000 samples of size 100 for 27 different sets of parameters: 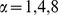, 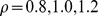, *F* being one of the three distributions mentioned above. Apart from the extensive study of [27], usual fluctuation experiment samples have size of order a few tens, which motivated my choice for the sample size. The range of values for *ρ* is typical of practical situations. For *α*, very small values were not considered as significant: if *α*<1, a large part of the information is contained in the frequency of zeros: the so called 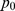-method gives almost as good results on *α* as any other estimator, independently from the model [14].

**Figure 2:**
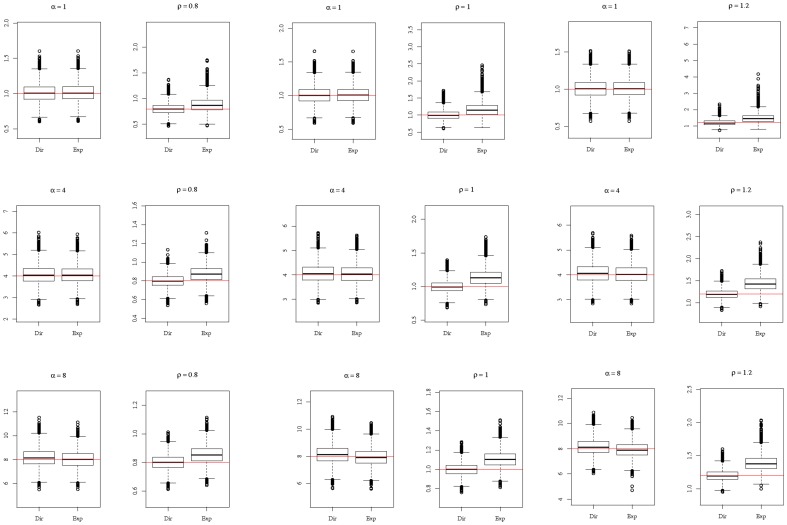
Boxplots of estimates of *α* and *ρ*, using the exponential and the Dirac models. Red horizontal lines mark true values of the parameters. For each of the 9 sets of parameters 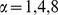 (rows) and 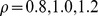 (columns), 10000 samples of size 100 of the 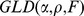 were simulated, *F* being the Log-normal distribution adjusted on Kelly and Rahn's data. The estimates of *α* and *ρ* were calculated with the two models Dirac and exponential. Each boxplot represents the distribution of the 10000 estimates obtained by the Dirac model (left) and the exponential model (right).

For each of the 270000 samples, and for the two models 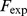 and 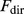, the estimates of *α* and *ρ* were computed, together with their confidence intervals at level 95%. The results obtained with the three distributions Gamma, Log-normal, and Inverse Gaussian, turned out to be very similar. Only the results for Log-normal division times are reported here. Figure 2 displays the boxplots of the estimated values of *α* and *ρ* for the 9 couples of parameters 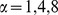, 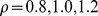. The following visual observations can be made:

- the classical exponential model clearly overestimates *ρ* and has a rather large dispersion of estimated values,
- the Dirac model correctly estimates *ρ*. It induces a much smaller bias and dispersion,
- both models correctly estimate *α*.

Further precisions are given in Table 1, were mean biases on 10000 samples are given for each of the 9 couples of parameters 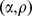 and the two models. The mean bias for estimates of *ρ* using the exponential model (last column of Table 1) is quite sizeable: between 10% and 30% of the true value.

**Table 1:** Mean biases on estimates of alpha and rho.

parameters	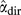	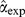	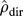	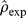
*α* = 1, *ρ* = 0.8	0.011	0.016	0.003	0.083
*α* = 1, *ρ* = 1.0	0.008	0.011	0.002	0.163
*α* = 1, *ρ* = 1.2	0.010	0.010	0.003	0.278
*α* = 4, *ρ* = 0.8	0.061	0.064	0.000	0.073
*α* = 4, *ρ* = 1.0	0.061	0.041	−0.001	0.137
*α* = 4, *ρ* = 1.2	0.067	0.033	−0.001	0.235
*α* = 8, *ρ* = 0.8	0.166	0.028	0.002	0.056
*α* = 8, *ρ* = 1.0	0.142	−0.059	−0.001	0.107
*α* = 8, *ρ* = 1.2	0.156	−0.079	0.000	0.188

For each of the 9 sets of parameters (left column), 10000 samples of size 100 of the 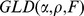 were simulated, *F* being the Log-normal distribution adjusted on Kelly and Rahn's data. The estimates of *α* and *ρ* were calculated with the two models Dirac and exponential. The estimated bias is the mean difference between the estimate and the true value. Biases on *ρ* with the classical model (rightmost column) are of order 10% to 20%.

The quality of confidence intervals when the model is not adapted is illustrated on Table 2. For each of the 27000 samples of size 100, confidence intervals for *α* and *ρ* at confidence level 95% have been computed using the exponential and Dirac model. Out of them, a theoretical proportion of 0.95 should contain the true value of the estimated parameter. The proportion of the 10000 intervals containing the true value has been computed for each value of the parameters. Table 2 shows the results for the Log-normal samples (results for the other two distributions are similar). The confidence intervals for *α* had a correct proportion of success for both models, slightly better for estimates using the exponential model. Confidence intervals on *ρ* using the Dirac model are also correct. However, the estimation of *ρ* using the exponential model was not reliable: up to 30% of the 95% confidence intervals did not contain the true value of *ρ* (last column of Table 2). This result is in accordance with the strong bias discussed above.

**Table 2:** Proportion of success for 95% confidence intervals.

parameters	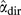	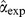	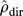	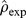
*α* = 1, *ρ* = 0.8	0.950	0.950	0.951	0.960
*α* = 1, *ρ* = 1.0	0.951	0.952	0.946	0.954
*α* = 1, *ρ* = 1.2	0.954	0.953	0.950	0.957
*α* = 4, *ρ* = 0.8	0.945	0.947	0.949	0.896
*α* = 4, *ρ* = 1.0	0.947	0.950	0.947	0.845
*α* = 4, *ρ* = 1.2	0.949	0.952	0.946	0.787
*α* = 8, *ρ* = 0.8	0.948	0.956	0.950	0.878
*α* = 8, *ρ* = 1.0	0.948	0.953	0.952	0.805
*α* = 8, *ρ* = 1.2	0.944	0.948	0.949	0.695

For each of the 9 sets of parameters (left column), 10000 samples of size 100 of the 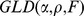 were simulated, *F* being the Log-normal distribution adjusted on Kelly and Rahn's data. The 95% confidence intervals for *α* and *ρ* were calculated with the two models Dirac and exponential. The entries of the table are proportions of the 10000 samples for which the true value is in the confidence interval. A result close to 0.95 indicates a satisfactory estimation.

**Figure 3:**
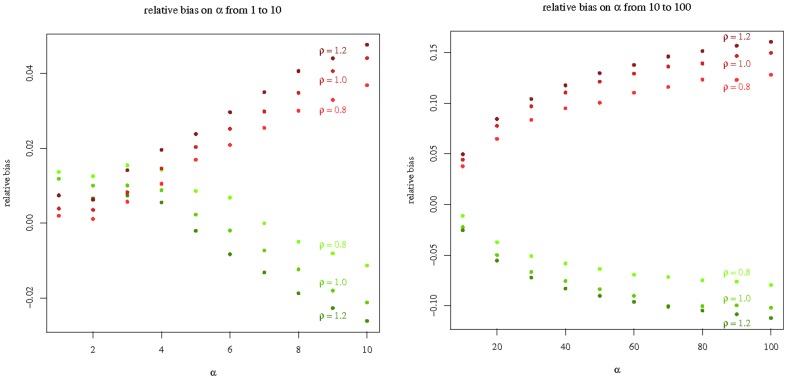
Relative bias on *α* between the exponential and the Dirac models. Ten thousand samples of size 100 were simulated for the 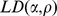 for alpha between 1 and 10 (left panel), then between 10 and 100 (right panel) and 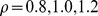. The estimate of *α* was computed using the 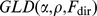, then averaged over all samples. The relative bias was calculated as the difference between the mean estimate and the true value of *α*, divided by the true value of *α*. Results are plotted as red points. The results for the opposite experiment (i.e. simulating the 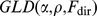, and estimating using the 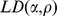) are plotted as green points.

The parameter of main interest being *α*, the results of Tables 1 and 2 are encouraging: the bias on *α* and the coverage probability of confidence intervals remain good, whichever model is used for estimation. In order to confirm this and evaluate the bias on *α* for larger values, another simulation experiment was made. For each of the two extreme models exponential and Dirac, for 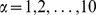 then 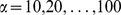 and 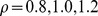, 10000 samples of size 100 were simulated, and the estimate of *α* calculated with the other model. It can be considered that the biases so obtained are an upper bound for the biases induced by using any of the two extreme cases for an unknown division time distribution. The relative bias was calculated as the difference between the mean estimate and the true value of *α*, divided by the true value of *α*. The results are plotted on Figure 3. For 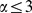, the bias is virtually negligible. For 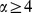, estimating as if division times were constant (red points) induces a positive bias, estimating as if they were exponential (green points) induces a negative bias. The relative bias remains smaller than 5% for 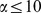. Notice that in all cases, for any given value of *α* the bias increases with *ρ*.

Having good estimates of the two parameters does not necessarily assure goodness-of-fit. In another experiment, 10000 samples of the 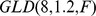 were drawn, *F* being the ‘realistic’ log-normal distribution. Each sample was adjusted both by the Dirac and exponential models: *α* and *ρ* were estimated for each model and the goodness-of-fit of the sample with the two adjusted distributions was tested, using the discrete version of the Kolmogorov-Smirnov test implemented in the R package dgof [28]. The test detected the difference in about 40% of the case (39% of p-values below 0.05 for the Dirac model, 43% for the exponential model). However, it must be observed that since the data were used to calculate the adjusted distribution, the p-values cannot be interpreted as if the distribution was independent from the data. More significantly, the comparison of Kolmogorov-Smirnov distances showed that the Dirac model was a better adjustment in 67% of the cases. This is coherent with the results of Table 1.

### Published data sets

The simulation study of the previous section indicates that the estimates of *α* should be coherent whatever the model, whereas the exponential model overestimates *ρ*. In order to evaluate the difference in actual experiments, five sets of published data were used. Luria and Delbrück [1] (Table 2, p. 504) had data under three different experimental conditions. I have grouped in sample A experiments numbers 1, 10, 11 and 21b; in sample B experiments 16 and 17. Data published in Boe et al. [27], Rosche and Foster [29], and Zheng [12] were also used. For each data set the 95% confidence intervals on *α* and *ρ* were computed using the exponential and the Dirac model. Results are reported in Table 3. The data set from [29] has a high frequency of zeros, and no jackpot; this explains why *ρ* cannot be reliably estimated by the exponential model. The Dirac model gives a more realistic estimate. In all cases, confidence intervals for *α* are similar. Confidence intervals on *ρ* are different, even though they overlap. As an example, for the Boe et al. data [27], the estimate of *ρ* given by the Dirac model is 0.738; the estimate given by the exponential model is 0.824, i. e. 11.6% larger. That difference is coherent with what has been observed on simulated data. Also, the amplitudes of the confidence interval under the Dirac and exponential models are 0.134 and 0.172: the precision under the Dirac model is better.

**Table 3:** Confidence intervals for published data sets.

reference	size	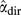	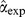	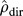	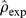
Luria & Delbrück A [1]	42	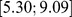	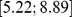	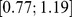	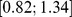
Luria & Delbrück B [1]	32	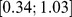	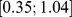	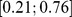	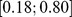
Boe et al. [27]	1102	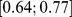	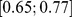	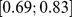	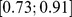
Roshe & Foster [29]	52	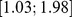	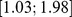	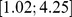	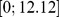
Zheng [12]	30	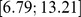	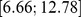	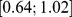	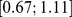

For 5 published data sets, the 95% confidence intervals on *α* and *ρ* were calculated with the two models Dirac and exponential.

The goodness-of-fit was tested for the two models, using the discrete version of the Kolmogorov-Smirnov test [28]. The results, reported in Table 4, are not conclusive: both adjustements are good in all cases. The Dirac model is (slightly) better for three datasets out of five.

**Table 4:** Kolmogorov-Smirnov goodness-of-fit tests for published data sets.

	Dirac model		Exponential model	
reference	distance	p-value	distance	p-value
Luria & Delbrück A [1]	0.055	1.000	0.057	0.999
Luria & Delbrück B [1]	0.069	0.998	0.055	1.000
Boe et al. [27]	0.015	0.955	0.006	1.000
Roshe & Foster [29]	0.046	1.000	0.049	1.000
Zheng [12]	0.063	1.000	0.070	0.997

The Kolmogorov-Smirnov distance between the sample and the adjusted distribution was calculated for the two models Dirac and exponential. The parameters of the adjusted models were estimated from the data by the GF method. Since the adjusted model used estimations from the data, the p-value can only be taken as an indication. Calculations were made using the R package dgof [28].

## Discussion

Dealing with fluctuation analysis experiments and the calculation of mutation rates, three different levels must be distinguished: the reality which remains unknown, the mathematical model, and the estimation technique.

### The unknown reality

Mutant counts at the end of a fluctuation analysis experiment are the result of

1. a random number of mutations occurring with small probability among a large number of cell divisions,
2. the random times during which mutant clones stemming from each mutation develop,
3. the number of cells that a clone developing for a given time may produce.

### The mathematical model

All models can be interpreted according to the same three points. The first two are hardly disputable; the third one is much more controversial.

1. Due to the law of small numbers, the number of mutations must follow a Poisson distribution with expectation *α*, understood as the mean number of mutations occurring during the experiment, i.e. the product of the individual probability of mutation (also called mutation rate) by the final number of cells.
2. The developing time of a random clone has exponential distribution with parameter *ρ*, provided the time scale has been chosen so that the growth rate of mutants is 1: *ρ* is the ratio of the growth rate of normal cells to that of mutants, or else the relative fitness.
3. The distribution of the number of cells that a clone developing for a given time can produce depends on various modelling hypotheses, such as:
  - if a mutation occurs during a division, only one of the two daughter cells is a mutant
  - mutant clones develop forever as mutants (no back mutation)
  - no cell dies before dividing
  - the division times are independent and identically distributed
  - the distribution of division times is exponential

Since the early forties (and maybe even before: see [30]), mathematicians have struggled to propose sets of modelling hypotheses that allowed explicit computations of probabilistic distributions. Following Lea and Coulson [5], Bartlett [6]), and Haldane [30], [31], the first four of the above hypotheses have been widely agreed upon. As for the distribution of division times, the exponential model that leads to the classical Luria-Delbrück distribution has largely prevailed [32], [33], though constant division times have also been considered [30], [31]. At first, only the case were normal cells and mutants had the same growth rate (*ρ* = 1) was studied. But soon, with Mandelbrot [7] and Bartlett [8], the model was generalized to differential growth rates [9]–[13]. Strangely enough, whereas the Poisson approximation (point 1. above) has been considered an obvious fact since Luria and Delbrück [1], the exponential distribution of development times (point 2.) has remained unnoticed, even though it was known as a basic fact of branching process theory at least since the sixties [34]. It was remarked in [14], and leads not only to a much simpler derivation of closed mathematical formulas, but also to simple and efficient simulation algorithms.

A distinctive hypothesis of the model considered here (as in most previous works), is that cells can only divide and never die. A model taking cell deaths into account was described in [35], and an estimation procedure was proposed. In practice, the proportion of deaths is known to be rather low [22], [36]. As shown in [35], neglecting cell deaths underestimates *α* and *ρ*. Another dubious hypothesis of the models considered so far is the independence of individual division times. The independence hypothesis was questioned very early [37]. Indeed, actual division time data show two types of correlation [38]: between the division times of a mother cell and its two daughters, and between the two sisters conditioning on the mother. It was remarked long ago by Powell [39] (see also [40], [41]) that sister-correlations do not influence exponential growth. The effect of mother-correlation on growth rates was discussed by Harvey in [41]. Its influence on the estimation of parameters in fluctuation analysis will be the object of future work.

### The estimation technique

From Luria and Delbrück [1], the mean number of mutations *α* has been the parameter of interest, whereas the relative fitness *ρ* was regarded at best as a nuisance parameter, or very often taken as fixed: *ρ* = 1 [2]–[4]. Indeed, the relative fitness can be independently estimated, by separately growing clones of mutants and normal cells, and calculating their growth rates [42]. If this has been done, then *ρ* can be considered as known, which leads to a better estimation of *α*, as pointed out in [14]. Yet *ρ* is rarely known in practice. Its independent calculation may be difficult in some cases (in vivo experiments for instance). Considering differential growth rates is necessary, as pointed out by several authors [9]–[14]; however, many studies are still being made using the LD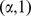 without questionning the equal rate hypothesis (e.g. [43], [44]).

Once a mathematical model has been chosen, many estimation procedures for *α* and/or *ρ* are available [4], [14]. As in any parametric estimation problem, the questions are:

- are estimates unbiased?
- can confidence intervals be computed?
- is the mean squared error minimal?

Only three methods answer positively the first two questions: the 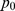-method [1], [4], the Maximum Likelihood (ML) method [12], [13], [45], [46], and the Generating Function (GF) method [14]. As in many other estimation problems, the best method in terms of mean squared error is the ML method. As was shown in [14], the 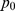-method performs well for small values of *α*. The GF method is nearly as precise as the ML, with a much broader range of applicability, and virtually null computing time.

To go further, three more criteria must be added:

- to how many models can the procedure be applied?
- can it work on a wide enough range of values of *α* and *ρ*?
- is it robust to variations of modelling hypotheses, or else how much bias estimating with a wrong model does induce?

As far as the first and third questions are concerned, the winner is the 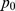-method: the distribution of the estimator is easily computed under any model, and the result does not depend on any hypothesis, except the fact that cells always divide and never die (see [35] for an alternative in the case of cell deaths). However, it relies upon a positive number of zeros in the sample, and is therefore limited to relatively small values of *α* (smaller than 2 in practice). Such a limitation is not statistically acceptable.

Regarding the first question, the ML procedure can be applied if the probabilities of mutant counts can be computed as a function of the parameters. This is the case for only two distributions so far: the classical 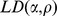 (independent exponential division times, no deaths), and the 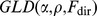 (constant division times, no deaths). The GF procedure can be applied to any 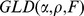, provided the distribution *F* has been previously estimated. It was applied to a cell-death model in [35]. Actually the Monte-Carlo algorithm proposed here can be used for any model, as soon as clones can be simulated. If the distribution of division times is unknown, any one of the two models above (exponential or constant division times) can be chosen.

The second question has been discussed in [14]. Even with a very careful algorithmic implementation [12], [47], the ML method can compute estimates only for samples in which the maximal value does not exceed a certain limit. Yet a crucial feature of mutant counts is the appearance of jackpots, i.e. unusually large values. For the ML method to be applied, the highest jackpots must be levelled out, which induces a systematic bias both on *α* and *ρ*. This explains why the ML method can be used only when large jackpots are very unlikely, or else if *α* is small enough and *ρ* large enough; as an indicative range of values, 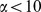 and 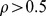 can be considered (admittedly, current experiments stay within that range).

Regarding the third question, estimating parameters with a wrong model can be expected to induce some bias, whichever estimation method is used. As shown in [14], the GF and ML methods output very similar results (when both can be used). So the conclusions of the ‘Results’ section would hold as well for ML estimates. The main question was to evaluate which bias could be expected from using either the Dirac or the classical exponential model, when data were simulated using a more realistic model. The estimation of *α* can be expected to be robust for low values, because when *α* is small, the information is concentrated on the first value 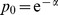 that depends only on *α*. The surprise was that it is still robust up to 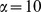, when 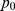 is very small and the 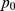-method cannot be used (Figure 3, left panel). For very large values of *α*, both models induce a bias on *α*, positive for the Dirac model, negative for the exponential model (Figure 3, right panel). The estimation of *ρ* is more sensitive to the model: estimating with the exponential model induces a positive bias; using the Dirac model reduces the bias (Figure 2 and Table 1).

## Models and Methods

### Division time distributions and growth rates

In this section, the probabilistic model of cell division and mutations is described, the relation between division times and growth rates is precised, and the goodness-of-fit of Kelly and Rahn's data [15] with three families of distributions is detailed.

In Kendall's notation [48], the model considered here is G/G/0:

- at time 0 a homogeneous culture of *n* normal cells is given;
- the division time of any normal cell is a random variable with distribution function *G*;
- when the division of a normal cell occurs, it is replaced by:
  one normal and one mutant cell with probability *p*,
  two normal cells with probability 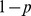;
- the division time of any mutant cell is a random variable with distribution function *F*;
- when the division of a mutant cell occurs, it is replaced by two mutant cells;
- all random variables and events (division times and mutations) are mutually independent.

**Figure 4:**
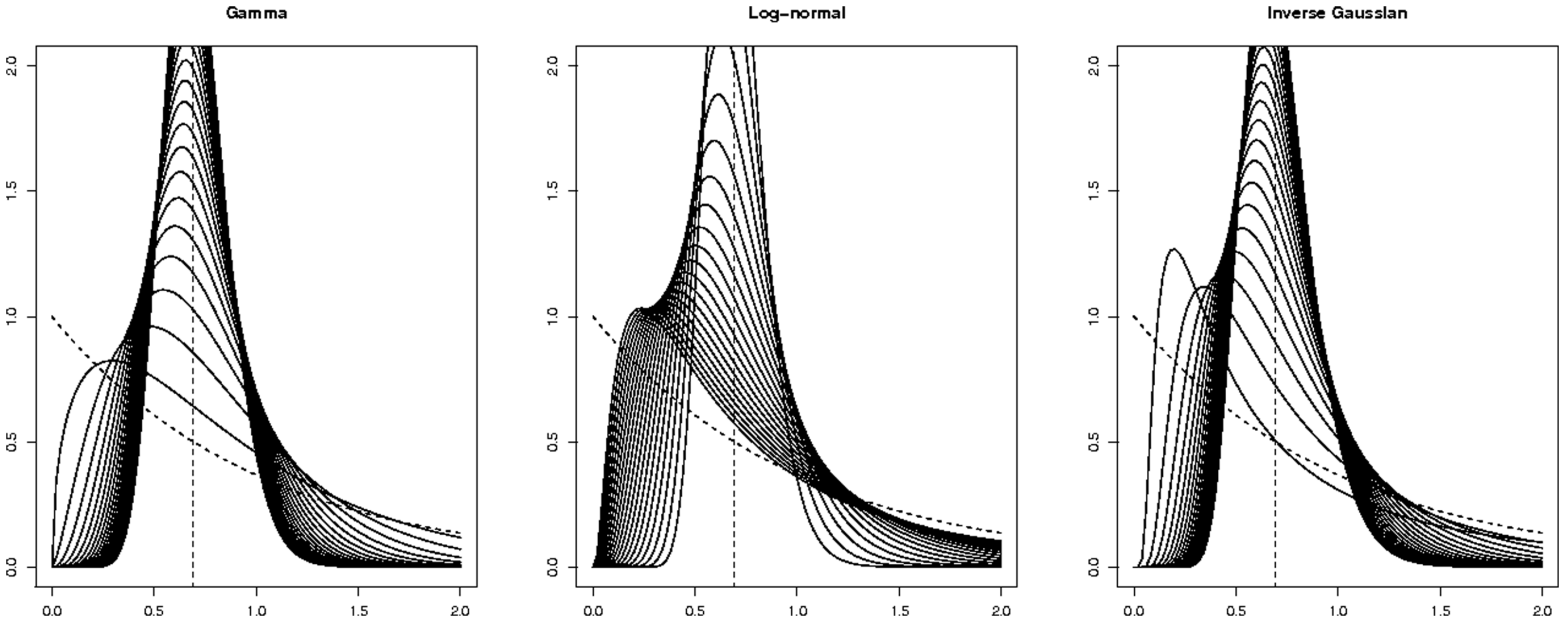
Densities of Gamma, Log-normal, and Inverse Gaussian. All densities have been rescaled to have unit growth rates. The dashed curve is the density of the exponential distribution with rate 1. The dashed vertical line locates the Dirac distribution at log 2.

The probabilistic results used here come from the theory of continuous time branching processes: see [49], [50]. To a distribution of division times corresponds an exponential growth rate for the corresponding clones: the growth rate of a clone with binary divisions is the point at which the Laplace transform of division times equals 1/2. If all division times are multiplied by a constant, the growth rate is divided by the same constant. Therefore scaling a distribution to have unit growth rate amounts to multiplying all division times by the initial growth rate. Here I assume that the time scale has been chosen so that the growth rate of normal cells is the relative fitness *ρ*, and the growth rate of mutants is 1:
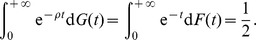
Two particular cases will be seen as extreme values for the distribution *F*: exponential and Dirac distributions.
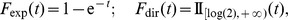
where 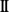 denotes the indicator function of an interval (1 or 0 according to whether the variable is in the interval or not). These distributions have coefficients of variation equal to 1 and 0 respectively. The coefficients of variation observed in experiments are of order 0.2 [23]. I have chosen three families of distributions to illustrate my results: Gamma, Log-normal and Inverse Gaussian. All three have the property to be invariant through scaling. For instance, if *X* has Gamma 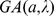 distribution, then *sX* has 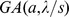 distribution; similar relations hold for the two other families. The probability distribution functions, Laplace transforms, and scaling parameters are given in Table 5. As many other families of distributions, these three encompass the two extremes of exponential and Dirac distributions as limit cases and interpolate between them. This is illustrated by Figure 4 where 20 densities of unit growth rate distributions are plotted for each family.

**Table 5:** Characteristics of three families of distribution.

Distribution	Gamma	Log-normal	Inverse Gaussian
parameters	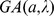	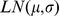	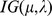
PDF	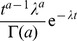	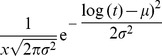	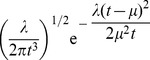
Laplace transform	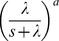	numeric	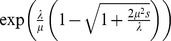
growth rate	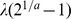	g.r. numeric	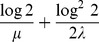
unit growth rate	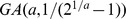	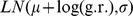	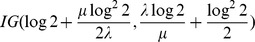

For Gamma, Log-normal and Inverse Gaussian distributions, the notation of parameters, the probability distribution function (PDF), the Laplace transform, the growth rate, and the scaling for unit growth rate are given.

**Figure 5:**
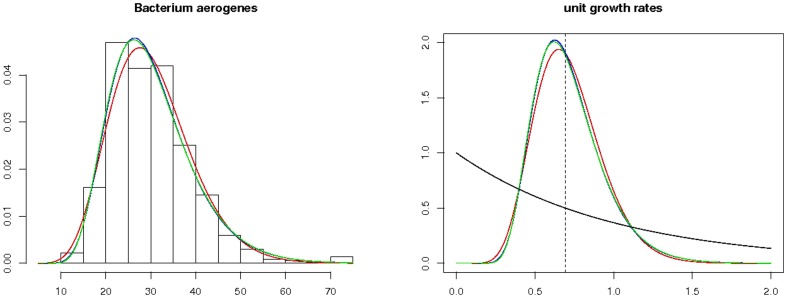
Adjusted distributions for Kelly and Rahn's data on Bacterium aerogenes [15]. On the left panel, the histogram of the data, and the three densities are superposed; the Gamma distribution appears in red, the Log-normal distribution in blue, the Inverse Gaussian in green. The blue and green curves are very close. On the right panel, the densities have been rescaled to unit growth rate. The dashed curve is the density of the exponential distribution, the dashed vertical line locates the Dirac distribution at log 2.

In order to get one realistic distribution per family, the historical observations of Kelly and Rahn on Bacterium aerogenes: Table 2 p. 149 of [15] were adjusted. A maximum likelihood estimation of the parameters on the data led to one particular distribution in each family, that was rescaled to unit growth rate. Figure 5 illustrates the fit. On the left panel, the histogram and the 3 densities are superposed; the right panel displays the corresponding densities after scaling to unit growth rate. Table 6 gives the parameters of the three densities, together with the p-values of the Kolmogorov-Smirnov and Anderson-Darling goodness-of-fit tests: all three fits turn out to be satisfactory.

**Table 6:** Adjusted distributions for Kelly and Rahn's data on Bacterium aerogenes [15].

Distribution	Gamma	Log-normal	Inverse Gaussian
parameters	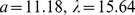	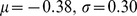	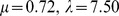
Kolmogorov-Smirnov	0.693	0.919	0.874
Anderson-Darling	0.505	0.852	0.813

A maximum likelihood estimation of the parameters on the data led to one particular distribution in each family, that was rescaled to unit growth rate. The parameters of the rescaled distribution are given, together with the p-values for the Kolmogorov-Smirnov and Anderson-Darling goodness-of-fit tests.

### Generalized Luria-Delbrück distributions

Consider an initial (large) number *n* of normal cells. Assume that the mutation probability *p* is small, that the time *t* at which mutants are counted is large, and that the asymptotics are such that the expected number of mutations *α* before time *t* is non null and finite. Using general results of branching process theory [49] and [50], it can be proved that the total number of mutants at time *t* approximately follows an integer valued distribution, whose probability generating function (PGF) is given by:
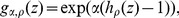
(1)with:
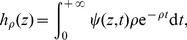
(2)where 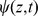 is the PGF of 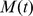, i.e. the number of cells at time *t* in a mutant clone, starting from one single cell at time 0.
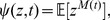
(3)where 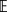 denotes mathematical expectation. The explicit expressions (1) and (2) are quite general, and do not depend on any modelling assumption apart from exponential proliferation. If the individual division times of mutants are supposed to be independent with common distribution *F*, then the function 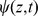 is uniquely defined in terms of *F*.

The interpretation of (1) and (2) is quite simple, and can be separated into the following two arguments.

- The number of mutations converges in distribution to the Poisson distribution with parameter *α* (this remark had already been made by Luria and Delbrück [1]). From each mutation stems a mutant clone that develops at final time *T* into a random number of mutants, each with PGF 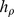. A random number of such clones must be added: the result is a Poisson sum of independent random variables with PGF 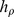. This yields equation (1).
- Any given mutation happens at some division instant chosen at random (i.e. uniformly distributed) among all division instants. Due to exponential growth, division instants are more concentrated near the end of the observation interval. It can be proved that the difference between the final time and a randomly chosen division instant, i.e. the developing time of a typical mutant clone, is exponentially distributed with parameter *ρ*. Therefore the size at final time of a typical mutant clone is an exponential mixture of sizes at fixed time *t*. Hence equation (2).

Precise mathematical statements and proofs of the asymptotics described above have been given in [14], and will not be reproduced here. I propose to name *Generalized Luria-Delbrück* distribution with parameters *α*, *ρ*, and *F* and denote by 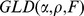, the probability distribution on the set of integers whose PGF 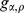 is defined by (1) and (2). Observe that it depends on the division time distribution of normal cells *G* only through the growth rate *ρ*, whereas it does depend on the actual division time distribution *F* of mutant cells. The particular case 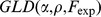 is the classical Luria-Delbrück distribution 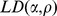. In that case,
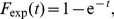
and
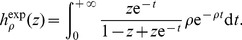
(4)The exponential case has been known for a long time: see Zheng [32], [33] for historical accounts. As shown in [14], formula (4) comes from the fact that the size of a mutant clone at time *t* follows the geometric distribution with parameter 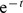, a fact already pointed out by Yule [51] (see also [50], p. 109). It turns out that explicit expressions of 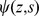, 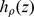, and 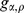 can also be given in the case where division times are constant, which is the object of the next section.

### Constant division times

Here it is assumed that division times of mutants are constant, i.e. *F* is the Dirac distribution at 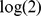 (to ensure unit growth rate).
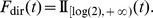
Thus the generalized Luria-Delbrück distribution 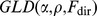 is considered. The idea can be traced back to Haldane who used it to propose an approximation heuristics for calculating the probabilities of mutant counts: see Sarkar [30] and Zheng [31] (actually, Haldane's model can be related to the particular case 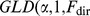).

For the 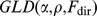, formula (2) becomes:
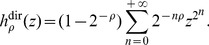
(5)To the best of my knowledge (5) is new. Here is how it is derived. With constant division times, say all equal to *a*, the population doubles at multiples of *a*. Hence the exponential growth rate is 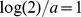, therefore 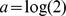. Between instants *na* and 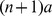, there are 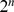 cells in the clone. Hence the generating function at time *s*:
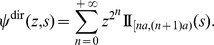
Integrating against the exponential distribution with parameter *ρ* gives:
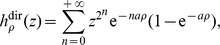
hence (5), since 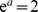.

Not only the PGF, but also the probabilities of the 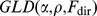 can be easily computed. Indeed, let 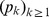 denote the probabilities of the distribution with PGF 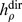:
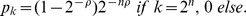
Let 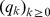 be the probabilities of the 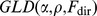. They can be computed by the following well known recursive formula, easily deduced from the probability generating function (1) (see [52] and references therein):
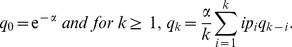
(6)The algorithm has been encoded in the R script available online: the probabilities, cumulated distribution function and quantile function of the 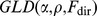 are provided. The log-likelihood and its derivatives with respect to the parameters also have explicit algorithms, almost identical to those implemented for the 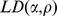 by Zheng [13]. The conclusion is that the estimation of *α* and *ρ* can be conducted for the 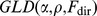 exactly as for the 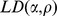, either by the classical Maximum Likelihood method [13] or by the generating function method [14]. The algorithms are even faster and numerically more stable in the constant division time model.

### General division times

**Figure 6:**
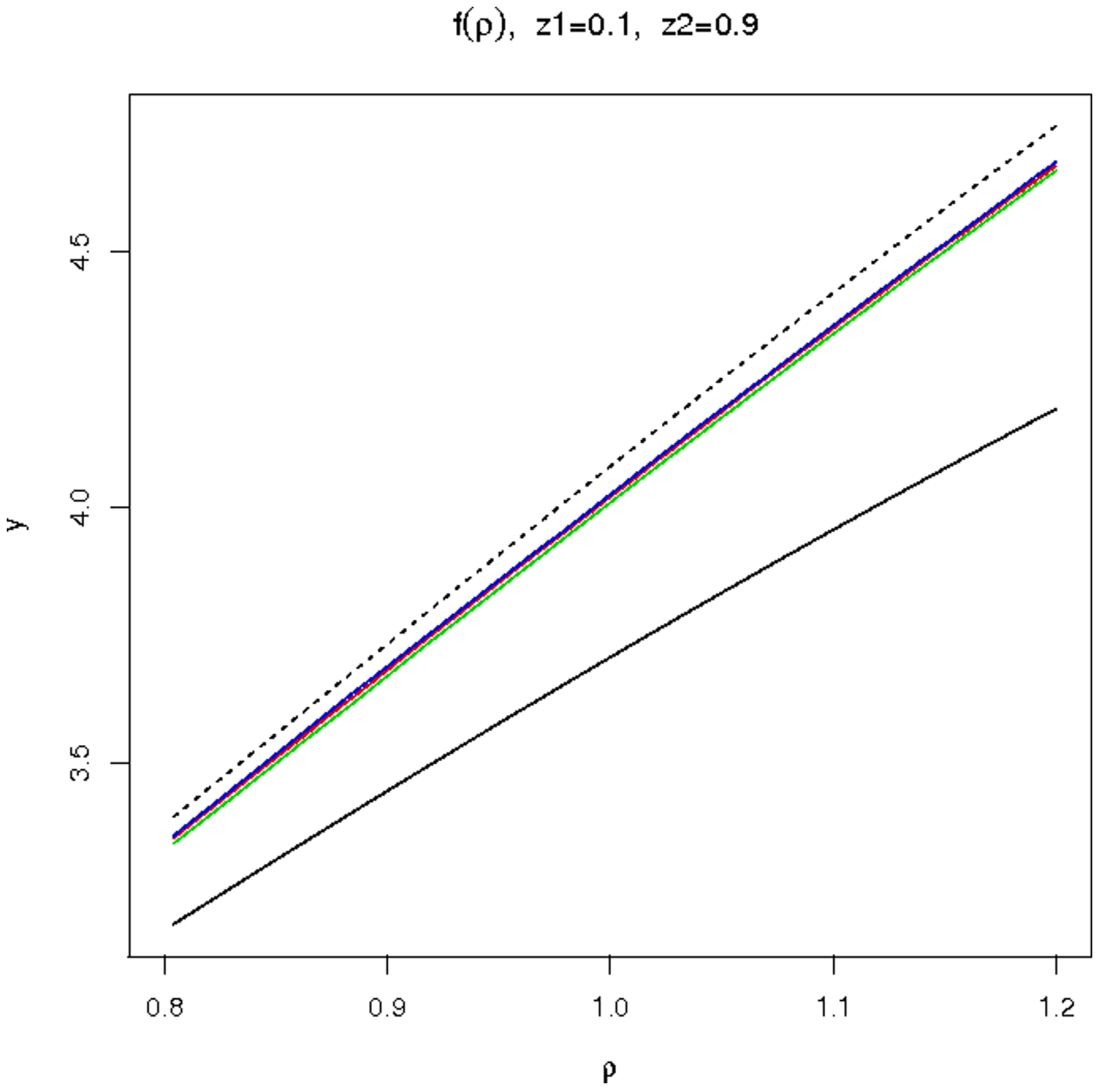
Ratios for GF estimators of the relative fitness *ρ*. Ratios 
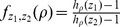
 as functions of *ρ*, for 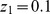 and 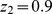. The ratios depend on the division time distribution: exponential (solid black), Dirac (dashed black), Gamma (red), Log-normal (blue), Inverse Gaussian (green). The realistic distributions are close together, and closer to the Dirac case than to the exponential case. This explains why the classical Luria-Delbrück model induces a positive bias on the estimation of *ρ*, and why the Dirac model yields better results.

No distribution *F* other than 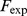 and 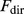 leads to such closed expressions as (4) and (5). However, it is possible to compute numerically 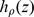 for any *F*, using a Monte-Carlo algorithm that will now be described. If a division time distribution is given, sequences of independent division times can be simulated at will. From such a sequence, a clone can be simulated up to any arbitrary time, outputing the number of cells as a function of time. That function of time is encoded by the sequence of instants at which the function increases by 1, i.e. when divisions occur. Choose a value 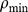, such that any subsequent evaluation of 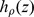 will be made for values of *ρ* larger than 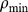. In simulations, I have chosen 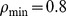, but this value could be adjusted. Let 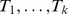 be *k* independent instants, simulated according to the exponential distribution with parameter 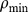. A crucial observation is that if 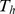 is exponentially distributed with parameter 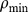, then for any 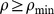, 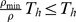 is exponentially distributed with parameter *ρ*. For 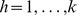, denote by 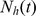 the number of living cells at time *t* in a random clone, starting from a single mutant cell at time 0, simulated up to time 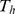. For any 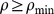, and any 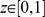, consider:
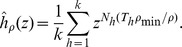
By the law of large numbers, as *k* tends to infinity, 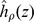 converges to 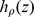. The central limit theorem yields a precision of order 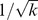 on the result. In simulations (in particular to compute the curves of Figure 6), *k* has been fixed to 10^5^. Observe that the (time consuming) simulation of the *k* clones needs to be done only once: from there, all subsequent evaluations of 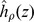 will be deduced.

As will be seen in the next section, the estimation of *α* and *ρ* and the computation of their confidence intervals require repeated evaluations of the derivative in *ρ* of 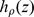. Using the procedure above to evaluate that derivative by finite differences would lead to quite unprecise results. Another procedure, similar to the previous one, is proposed instead. The derivative in *ρ* of 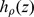 is:
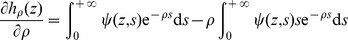


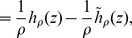
with:
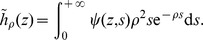
Now 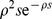 is the density of the Gamma distribution 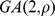 (sum of two independent exponentially distributed random variables). Therefore 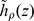 is the PGF of the number of cells in a clone starting from a single mutant cell at time 0, observed up to an independent, Gamma distributed random time. Let 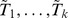 be *k* independent instants, simulated according to the Gamma distribution with parameters 2 and 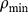. For 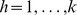, denote by 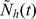 the number of living cells at time *t* in a random clone, starting from a single mutant cell at time 0, simulated up to time 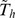. For any 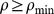, and any 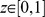, consider:
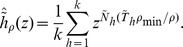
By the law of large numbers, as *k* tends to infinity, 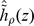 converges to 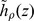.

Further savings in computer time can be obtained by the following remark. Let 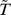 follow the Gamma distribution with parameters 2 and *ρ*. Let *U* be another random variable, independent from 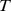, uniformly distributed on the interval [0,1]. Then *UT* follows the exponential distribution with rate *ρ*. Therefore the same *k* clones, simulated up to Gamma distributed instants, can be used to estimate the values of both 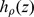 and 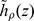.

### Generating function estimators

The main goal of fluctuation analysis is to estimate the mutation probability *p*, from a sample of mutant counts. If an estimate of the mean number of mutations *α* has been calculated, then an estimate of *p* can be deduced, dividing by the final number of cells: the parameter of main interest is *α*. Many methods of estimation for *α* have been proposed: see [4]. The simplest consists in estimating the probability of observing no mutant: 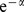; this is the original method used by Luria and Delbrück [1], and is usually referred to as “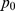-method”. Observe that the result does not depend on *ρ*, nor on *F*. Therefore the 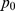-method is completely independent from any modelling hypothesis. It that sense it is the most robust of all methods. However, the 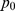-method can be used only if *α* is small enough (so a sizeable number of tubes do not contain any mutant). As explained in [14] and in the discussion section, such a limitation cannot be accepted.

Apart from the 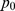-method, any other consistent estimator of *α* must depend on the value of *ρ* and on the mutant division time distribution *F*. Maximum Likelihood is usually considered the best estimation method in a parametric inference problem. For the estimation of the parameters *α* and *ρ* of the classical 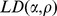, it has been recommended by several authors: [12], [13], [45], [46]. In [14], its limitations were pointed out, and an alternative procedure, based on the empirical probability generating function (EPGF), was proposed. It turns out that the EPGF method can be adapted to the general case of the 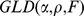, whereas the Maximum Likelihood cannot. It only relies upon the numerical evaluations of 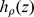 and its derivative in *ρ*. For the 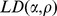 and 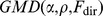 explicit formulas are available, for the other cases a Monte-Carlo algorithm was described in the previous section. The procedure is described below, and the reader is refered to the R functions that have been made available online for implementation details: they include estimation, confidence intervals, and hypothesis testing.

Let 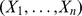 be a sample of independent random variables, each with 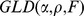 distribution. Recall the probability generating function of the 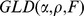:
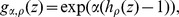
with:
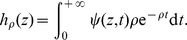
Define the empirical probability generating function (EPGF) 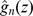 as:
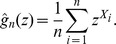
The random variables 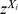 are bounded and mutually independent: by the law of large numbers, 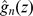 is a consistent estimator of 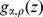, for any *z* in [0,1]. For 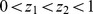, consider the following ratio:
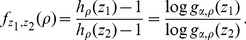
(7)The function that maps *ρ* onto 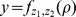 is continuous and strictly monotone, hence one-to-one. Therefore the inverse, that maps *y* onto 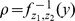, is well defined. For 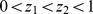, let 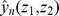 denote the following log-ratio.
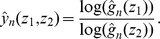
An estimator of 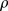 is obtained by:
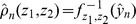
Then an estimator of *α* by:
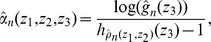
where 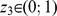 is a new control, possibly different from 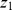 and 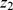. Observe that 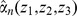 depends on 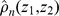, whereas 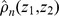 only depends on the arbitrary choice of the couple 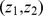. They will be referred to as generating function (GF) estimators. The strong consistence and asymptotic variance of the GF estimators were studied in [14], and mathematical details will not be reproduced here. In particular, Proposition 4.1 of that reference gives the explicit form of the asymptotic covariance matrix, upon which inference procedures are based (confidence intervals and hypothesis testing). The asymptotic covariance matrix has been encoded in the *R* functions made available online; it expresses in terms of:

- the PGF 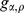 evaluated at 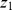, 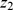, 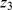, and their products two by two,
- the PGF 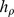 evaluated at 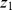, 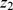, 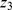,
- the derivative in *ρ* of 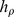, evaluated at 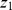, 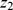, 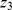.

The GF estimators depend on the three arbitrary values of 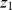, 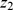 and 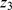. Another tuning parameter has to be added. In the 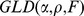 the parameter *ρ*, determines the size and frequency of much larger values than usual (called “jackpots” in [1]). For 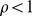, some very large values can be obtained, even for a small *α*. Using the empirical probability generating function is a simple way to damp down jackpots, and get robust estimates. The variable *z* can be seen as a tuning parameter for the damping. At the limit case 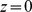, 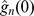 is simply the frequency of null values, and 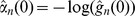 is the so called 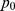-estimator of *α*, already proposed in [1] (it does not depend on *ρ* nor *F*). For 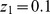, only small observations will be taken into account, whereas for 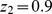, much larger values will influence the sum. Thus the empirical probability generating function damps down jackpots in a differential way according to 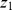 and 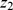. Choosing 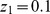 and 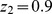 will contrast small values compared to jackpots, which explains why 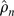 can efficiently estimate *ρ* for small *α*'s. However, for large values of *α* (say 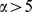), even 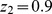 will output very small values, below the machine precision. This will make the estimates numerically unstable. A natural way to stabilize them is to rescale the sample, dividing all values by a common factor *b*. This amounts to replacing *z* by 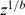 in the definition of 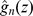:
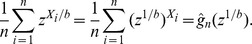
I proposed to set *b* to the *q*-th quantile of the sample, where *q* is another control. Based on simulation evidence, my best compromise is 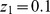, 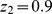, 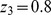, 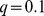. In the implementation of the GF estimators, the scaling factor *b* is set to the *q*-th quantile of the sample, and all data are divided by that scaling factor (which amounts to replacing 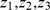 by 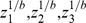). The estimators 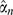 and 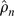 are computed with these values.

The GF estimators crucially rely upon the inverse of the function *f*, defined by (7). Figure 6 shows variations of *f* according to the underlying model. On that figure, plots of *f* for the exponential and Dirac model have been represented, together with plots of *f* for the 3 distributions determined by fitting actual data. The curves corresponding to realistic distributions are close together, and closer to the Dirac case than to the exponential case. From this graphics, it can be anticipated that estimating *ρ* with the classical Luria-Delbrück model induces a positive bias; this was indeed observed on simulations. Also the fact that the slope of the curve corresponding to the exponential model is smaller explains why the precision on *ρ* obtained by the classical method is worse.
